# Microdystrophin Gene Addition Significantly Improves Muscle Functionality and Diaphragm Muscle Histopathology in a Fibrotic Mouse Model of Duchenne Muscular Dystrophy

**DOI:** 10.3390/ijms24098174

**Published:** 2023-05-03

**Authors:** Viktorija Cernisova, Ngoc Lu-Nguyen, Jessica Trundle, Shan Herath, Alberto Malerba, Linda Popplewell

**Affiliations:** 1 Department of Biological Sciences, School of Life Sciences and the Environment, Royal Holloway University of London, Egham, Surrey TW20 0EX, UK; viktorija.cernisova.2013@live.rhul.ac.uk (V.C.);; 2 National Horizons Centre, Teesside University, Darlington DL1 1HG, UK

**Keywords:** Duchenne muscular dystrophy, muscle pathology, AAV8-microdystrophin, fibrosis

## Abstract

Duchenne muscular dystrophy (DMD) is a rare neuromuscular disease affecting 1:5000 newborn males. No cure is currently available, but gene addition therapy, based on the adeno-associated viral (AAV) vector-mediated delivery of microdystrophin transgenes, is currently being tested in clinical trials. The muscles of DMD boys present significant fibrotic and adipogenic tissue deposition at the time the treatment starts. The presence of fibrosis not only worsens the disease pathology, but also diminishes the efficacy of gene therapy treatments. To gain an understanding of the efficacy of AAV-based microdystrophin gene addition in a relevant, fibrotic animal model of DMD, we conducted a systemic study in juvenile *D2.mdx* mice using the single intravenous administration of an AAV8 system expressing a sequence-optimized murine microdystrophin, named MD1 (AAV8-MD1). We mainly focused our study on the diaphragm, a respiratory muscle that is crucial for DMD pathology and that has never been analyzed after treatment with AAV-microdystrophin in this mouse model. We provide strong evidence here that the delivery of AAV8-MD1 provides significant improvement in body-wide muscle function. This is associated with the protection of the hindlimb muscle from contraction-induced damage and the prevention of fibrosis deposition in the diaphragm muscle. Our work corroborates the observation that the administration of gene therapy in DMD is beneficial in preventing muscle fibrosis.

## 1. Introduction

Duchenne muscular dystrophy (DMD) is the most prevalent muscular dystrophy, affecting ~1 in 5000 boys [1]. It is an X-linked, progressive muscle disorder arising from mutations in the *DMD* gene, which normally codes for the dystrophin protein [2]. The loss of dystrophin interrupts the link between the cytoskeleton and the extracellular matrix (ECM), disrupting fiber integrity and causing the muscle to become prone to damage [3]. Persistent cell damage, in turn, leads to the aberrant deposition of ECM components and, thus, skeletal muscle fibrosis. The most promising treatments aim to restore dystrophin expression. Four FDA-approved drugs are now on the market for exon skipping, based on antisense therapeutics to target specific dystrophin exons to restore the reading frame of dystrophin and so the generation of a shorter, but partially functional protein. However, this approach is applicable to subgroups of patients and the efficacy is significantly hampered by the poor delivery of the antisense therapeutics [4]. Microdystrophin gene addition using adeno-associated viral (AAV) vectors is currently under testing in a number of clinical trials [5]. Furthermore, systems to improve the efficacy of these gene therapy applications are currently in preclinical development [6,7]. Most of these studies have been preclinically tested in the *mdx* mouse model that does not recapitulate the disease progression observed in patients [8], as muscle fibrosis, with the notable exclusion of the diaphragm, is only developed in aged mice. By crossing the *mdx* mouse with the *Dba2*/*J* model, the *D2.mdx* is produced that harbors the same mutation on the dystrophin gene as the *mdx* mouse, but is associated with an increased severity of muscle pathology [9,10,11]. While *mdx* mice have been used extensively to test new or established therapies for DMD, they do not recapitulate all of the features of the disease, and in particular, they do not show the fibroadipogenic tissue replacement that is observed in DMD patients. In comparison to *mdx* mice, *D2.mdx* present with an earlier disease onset and a more severe dystrophic phenotype [10]. In addition to this, *D2.mdx* mice have been shown to exhibit muscle degeneration as early as 3 weeks of age [12], suggesting that early intervention may provide a beneficial effect. The severity of the *D2.mdx* mouse has been attributed to a polymorphism in the latent TGF-β-binding protein 4 (LTBP4) [13]. LTBP4 is an ECM protein that binds to the latent form of TGF-β, thereby keeping it in its inactive form [14]. The deletion of a 12 amino acid sequence in the LTBP4, however, makes it more susceptible to proteolytic cleavage [15], thereby increasing TGF-β signaling [13] and, in turn, TGF-β-mediated fibrosis development and the inhibition of myogenesis [16,17,18,19]. This *D2.mdx* mouse model has been previously treated with either an AAV9 vector delivering a microdystrophin carrying the binding site for nNOS [20], or with microutrophin, which is homologous for microdystrophin [21], and long-term pathological benefits were reported for both. We show here for the first time that improvements in animal exercise endurance, muscle pathology, and function in the *D2.mdx* mouse model can be obtained with the early treatment of AAV8-MD1. Unlike the construct used by [20], MD1 contains hinge regions 1, 2, and 4, does not encode the nNOS binding domain, is packaged in an AAV8 capsid, and mirrors the agent being used in the GNT-004 clinical trial (EudraCT #2020-002093-27). Furthermore, our study uses an AAV dose of 4 × 10^12^ vg/mouse, which is about five times lower than the 2 × 10^13^ vg/mouse used by [20]. While in [20], the beneficial effect of AAV-microdystrophin was nicely shown in some skeletal muscles (i.e., tibialis anterior and extensor digitorum longus), we focused our study on the impact of AAV8-MD1 at the recognized peak of fibrosis in the *D2.mdx* mouse in the diaphragm, a respiratory muscle strongly affected by fibrosis and primarily involved in the development of the disease. While confirming the efficacy of the AAV8 serotype for the transduction of muscle and the therapeutic functionality of the GNT-004-like microdystrophin, this work additionally highlights the benefit of gene therapy treatment for the prevention of muscle damage and establishment of fibrosis in DMD.

## 2. Results

### 2.1. Administration of AAV8-MD1 Improves the Physical Abilities of D2.mdx Mice

**Figure 1:**
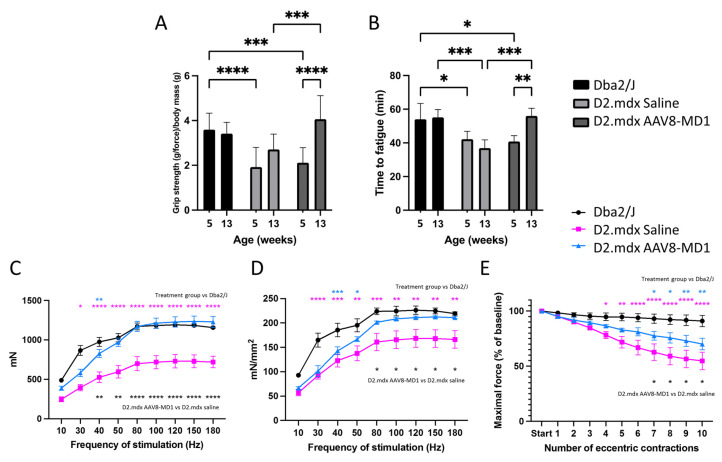
AAV8-MD1 administration greatly enhances physical abilities and muscle contractility properties in *D2.mdx* mouse. Male *Dba2/J*-positive control and *D2.mdx* mice were treated with a single IV injection of either saline or AAV8-MD1 at 4 × 10^12^ vg/mouse at 6 weeks of age. (**A**,**B**) Both forelimb grip strength (**A**) and time to fatigue (**B**) of AAV-treated *D2.mdx* mice showed a significant improvement at the end of the treatment with values that were normalized to the level of *Dba2/J*. (**C**) At 14 weeks of age, mice were placed under terminal anesthesia and maximal tetanic force of TA muscles at increasing frequency of stimulation was measured in situ. (**D**) Specific maximal force was calculated by normalizing the maximal force to the muscle cross-sectional area. (**E**) Resistance to eccentric contraction of muscles was evaluated and expressed as maximal force generated after each eccentric contraction as a percentage of the initial force. Data shown as means ± SEM, *n* = 4–5 mice. Statistical analysis was performed using two-way ANOVA followed by Tukey’s multiple comparisons test: *p* < 0.05 (*), *p* < 0.01 (**), *p* < 0.001 (***), *p* < 0.0001 (****).

To assess the efficacy of AAV8-microdystrophin (AAV8-MD1) in mice presenting consistent fibrosis of skeletal muscles, *D2.mdx* males were treated by tail vein injection with 4 × 10^12^ vg/mouse of AAV8-MD1 in sterile saline (*n* = 5), or with volume-matched saline only (*n* = 5), at 6 weeks of age. This is equal to ~2.5 × 10^14^ vg/Kg, a dose comparable to the current one used in clinical trials. Age- and sex-matched *Dba2/J* mice were injected with saline only (*n* = 4) and used as positive controls for all analyses. The skeletal muscles of these mice at this age already show a substantial amount of fibrosis, which makes this model more relevant than the *mdx* mouse generated on the *C57BL/10* background [22]. During the study, body mass was monitored on a weekly basis (Supplementary Figure S1A). The assessment of initial body mass at 6 weeks of age showed that the *Dba2/J*-positive control mice weighed significantly more than the *D2.mdx* mice at this age (*p* < 0.05). All mice grew consistently, and the final body mass was not significantly different between the *Dba2/J* and *D2.mdx* mice or between the two *D2.mdx* treatment groups (*p* > 0.05). The diaphragm, tibialis anterior (TA), soleus, and quadriceps were weighed after collection. Following the AAV-MD1 treatment, the weight of the diaphragm was normalized to the level of *Dba2/J*, while no significant difference was observed for the other muscles (Supplementary Figure S1B). Forelimb grip strength and fatigue resistance assessments were performed during the study to evaluate the efficacy of the treatment. Forelimb grip strength measurements were taken at 5 weeks of age (pre-treatment) and at 13 weeks of age (upon completion of the treatment regimen). The grip strength was recorded and normalized to the total body mass of the mouse (Figure 1A). No change in the forelimb grip strength was observed between 5 weeks and 13 weeks of age in healthy *Dba2/J* mice (5 weeks: 3.59 ± 0.212 vs. 13 weeks: 3.42 ± 0.146) or in *D2.mdx* mice treated with saline (5 weeks: 1.88 ± 0.174 vs. 13 weeks: 2.64 ± 0.111). Treatment with AAV8-MD1, however, resulted in a two-fold increase in grip strength post treatment compared to the starting point (5 weeks: 2.12 ± 0.179 vs. 13 weeks: 4.06 ± 0.27; *p* < 0.0001).

Fatigue resistance assessment was measured based on the time to fatigue on a treadmill with progressive speed escalation. The running time was recorded at 5 weeks of age (pre-treatment) and at 13 weeks of age (at completion of treatment regimen) (Figure 1B). No change in the time to fatigue was observed between 5 and 13 weeks of age in the *Dba2/J* mice (5 weeks: 54 min ± 4.71 min vs. 13 weeks: 55.2 min ± 2.3 min) or in the *D2.mdx* mice treated with saline (5 weeks: 39.2 min ± 2.24 min vs. 13 weeks: 40.8 min ± 1.89 min). In addition, at 13 weeks of age, the saline-treated *D2.mdx* mice performed significantly worse compared to the positive controls (*p* < 0.05). Conversely, an improved running time between pre- and post-treatment was observed in the AAV8-MD1-treated *D2.mdx* mice (5 weeks: 40.8 min ± 1.55 min vs. 13 weeks: 56.1 min ± 2 min; *p* < 0.01). Furthermore, this treatment resulted in significantly better time to fatigue compared to the saline-injected *D2.mdx* mice (*p* < 0.001), and their running times at the end of the experiment were no different to those of the *Dba2/J* mice.

At week 14, before harvesting the muscle samples, the mice were placed under terminal anesthesia and the maximal tetanic force for both TA muscles at an increasing frequency of stimulation was measured in situ. The maximal tetanic force of the left and right TA muscles per mouse was averaged (Figure 1C). At a frequency of stimulation of 50 Hz or above, the mice treated with AAV8-MD1 showed the same absolute force detected in *Dba2/J* mice (*p* < 0.05), while the saline-treated *D2.mdx* group generated significantly less force compared with both *Dba2/J* and AAV8-MD1-treated *D2.mdx* mice. The maximal force was normalized to the cross-sectional area (CSA) of the TA muscle to obtain the specific force (Figure 1D). Again, above 50 Hz stimulation, the specific force generated by the muscles of AAV8-MD1 mice was comparable with that of *Dba2/J* mice, suggesting that microdystrophin overexpression completely normalized the muscle strength in TA muscles. The resistance to eccentric contraction-induced muscle damage in TA muscles was additionally assessed (Figure 1E). Compared to *Dba2/J* mice, the *D2.mdx* mice treated with saline performed significantly worse after 4 (*p* < 0.05) and up to 10 eccentric contractions (from *p* < 0.01 to *p* < 0.0001 depending on the contraction). The administration of AAV8-MD1 resulted in 73 ± 5% of the maximal force of the initial reading following nine contractions, which was found to be no different to that of the *Dba2/J* mice. These data clearly demonstrate the beneficial effect of AAV8-MD1 systemic administration in *D2.mdx* mice. This vector was able to normalize most of the analyzed functional parameters to the wild-type levels.

### 2.2. Single Intravenous Administration of AAV8-MD1 Results in Significant Microdystrophin Protein Expression in the Diaphragm of D2.mdx Mice

**Figure 2:**
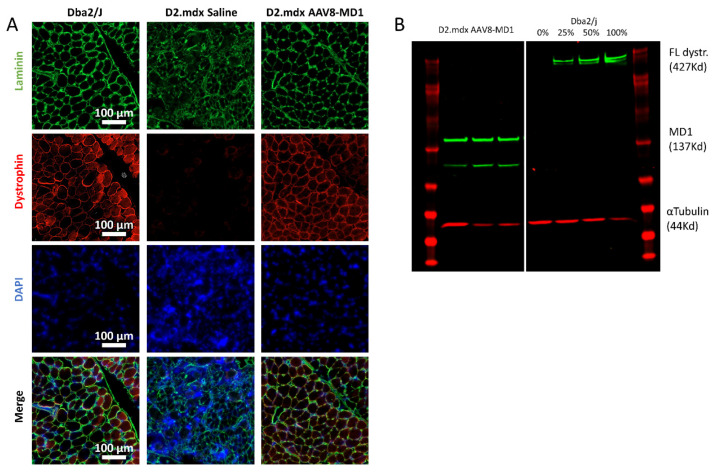
Microdystrophin protein expression in *D2.mdx* mouse diaphragms following systemic AAV8-MD1 treatment. Male *Dba2/J*-positive control and *D2.mdx* mice were treated with a single IV injection of either saline or AAV8-MD1 at 4 × 10^12^ vg/mouse at 6 weeks of age. (**A**) Representative images of muscle sections stained for dystrophin, laminin, and DAPI are shown for each treatment group (scale bar: 100 µm). (**B**) Western blot analysis demonstrates the level of microdystrophin expression. A standard curve for 0, 25, 50, and 100% of full-length dystrophin expression was created using a mixture of total protein from *Dba2/J* control and *D2.mdx* saline-treated mice. The level of microdystrophin was normalized to the loading control, α-tubulin, and expressed as a percentage of *Dba2/J* full-length dystrophin expression.

Respiratory failure has been a leading cause of loss of quality of life and contributes to death in DMD patients. We therefore focused on the assessment of AAV8-MD1 efficacy in the diaphragm following the treatment of the *D2.mdx* mouse, since this muscle closely recapitulates patients’ pathology. At the end of the experiment, the diaphragms were harvested and their weights were normalized for the body weights. The muscle mass of the diaphragm in the *D2.mdx* saline-injected mice was significantly higher compared to that of the *Dba2/J* mice (*p* = 0.012), while the administration of AAV8-MD1 significantly decreased the muscle mass of the diaphragm compared to the saline-injected *D2.mdx* group (*p* = 0.012), and its value was normalized to that of the *Dba2/J* control mice. The diaphragms were then sectioned and stained for dystrophin, laminin, and DAPI (Figure 2A). The diaphragm muscle fibers stained positive for dystrophin along the sarcolemma were counted and expressed as a percentage of dystrophin-positive fibers over the total number detected using laminin staining. The diaphragms of mice treated with AAV8-MD1 showed 76 ± 6.96% of dystrophin-positive muscle fibers. No dystrophin-positive fibers were detectable in the diaphragms from saline-treated *D2.mdx* mice. To further quantify the amount of dystrophin protein, total protein was extracted from the diaphragms and a Western blot analysis for dystrophin expression was performed (Figure 2B). The expression of microdystrophin in the *D2.mdx* mice treated with AAV8-MD1 was found to be 69.8% ± 22.5% of full-length dystrophin expression. As expected, no dystrophin-positive fiber or dystrophin-positive Western blot product was detected in the saline-injected *D2.mdx* muscle.

### 2.3. Administration of AAV8-MD1 Improves Diaphragm Histopathology in D2.mdx Mice

**Figure 3:**
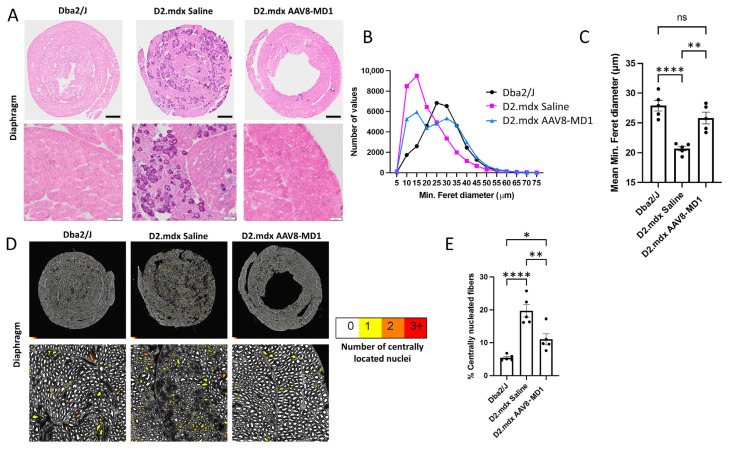
Effect of AAV8-MD1 on diaphragm histopathology of *D2.mdx* mice. Male *Dba2/J*-positive control and *D2.mdx* mice were treated with a single IV injection of either saline or AAV8-MD1 at 4 × 10^12^ vg/mouse at 6 weeks of age. (**A**) Mouse diaphragms harvested at 14 weeks of age were sectioned and stained with hematoxylin and eosin. Representative diaphragm sections were chosen for each sample group, showing whole diaphragm sections (top row; scale bar: 500 µm) as well as a close-up of each section (bottom row; scale bar: 100 µm). (**B**) Frequency distribution of the minimum Feret’s diameter of myofibers, calculated by Fiji/MuscleJ, and (**C**) subsequent analysis of the mean minimum Feret’s diameter suggest a clear improvement in myofiber size after AAV8-MD1 administration. (**D**) Cartography images of representative diaphragm sections for each sample group show whole diaphragm sections (top row) as well as a random close-up of each section (bottom row). For each fiber, the amount of centrally located nuclei was analyzed and the fibers were color-coded to represent this (white = 0, yellow = 1, orange = 2, red = 3+) (scale bar top row: 500 µm, bottom row: 100 µm). (**E**) The amount of centrally nucleated fibers was calculated and expressed as a percentage of total fibers. In C and E, data are shown as mean ± SEM, *n* = 4–5. Statistical analysis was performed using one- way ANOVA followed by Tukey’s multiple comparisons test: *p* < 0.05 (*), *p* < 0.01 (**), *p* < 0.0001 (****).

Hematoxylin and eosin staining revealed that the diaphragm from saline-injected *D2.mdx* mice displayed more substantial deterioration in the muscle architecture and quality and necrosis/inflammation with apparent calcification [22] compared to the *Dba2/J* muscle (Figure 3A). In contrast, the animals treated with AAV8-MD1 exhibited a muscle histology similar to that of *Dba2/J* mice, with fibers that were larger and more homogeneous in size. In order to assess the effect of AAV8-MD1 in terms of myofiber size, the mouse diaphragms were stained for laminin and were subsequently analyzed using Fiji/MuscleJ to calculate the minimum Feret’s diameter. The frequency distribution of the fibers’ diameter showed a clear negative skew in the frequency distribution of fibers in the saline-injected *D2.mdx* compared to the *Dba2/J* group, suggesting a higher proportion of small fibers in the *D2.mdx* muscles (Figure 3B). However, treatment with AAV8-MD1 resulted in a frequency distribution more similar to that of *Dba2/J*. Furthermore, the average minimum Feret’s diameter showed that the *D2.mdx* saline-injected control group had a mean diameter significantly lower than the positive *Dba2/J* control group (*p* < 0.0001) (Figure 3C). Treatment with AAV8-MD1 significantly increased the mean diameter compared to the saline-injected *D2.mdx* control group (*p* = 0.002) and normalized its value to that of the *Dba2/J* diaphragm fibers. Fiji/MuscleJ was also used to assess the amount of centrally nucleated fibers in sections stained for DAPI and laminin. The amount of centrally nucleated fibers, as shown in the cartography of the muscle sections (Figure 3D), calculated as a percentage of the total fiber count, confirmed that saline-treated *D2.mdx* mice had a significantly larger percentage of centrally nucleated fibers compared to *Dba2/J* mice (*p* < 0.0001), while the administration of AAV8-MD1 significantly inhibited the formation of centrally nucleated fibers compared to the *D2.mdx* saline-injected diaphragms (*p* = 0.0034) (Figure 3E).

### 2.4. Microdystrophin Gene Addition in D2.mdx Mice Prevents Fibrotic Tissue Deposition in Diaphragm Muscle

**Figure 4:**
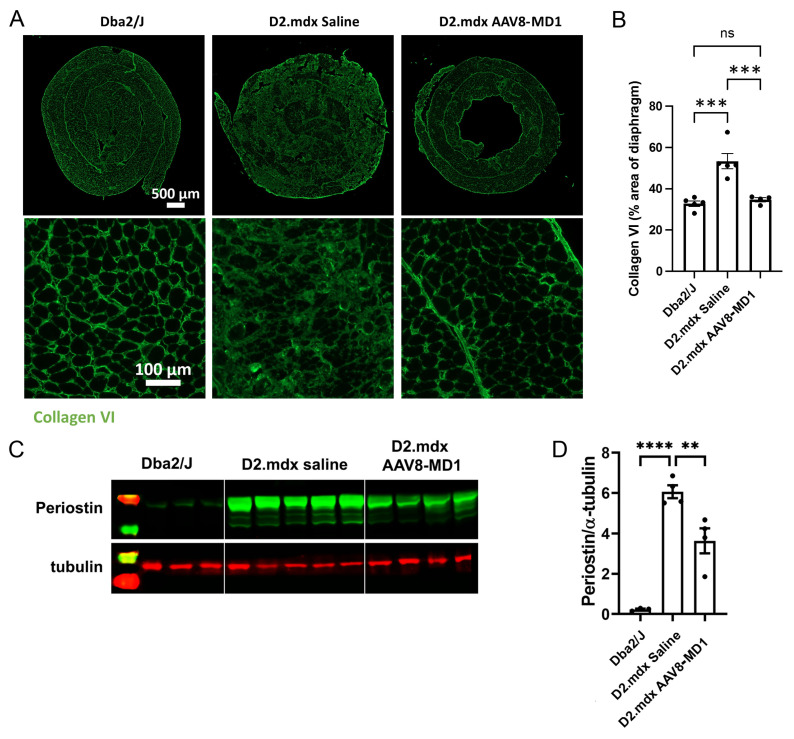
AAV8-MD1 delivery significantly reduces collagen VI and periostin expression in the diaphragm of *D2.mdx* mice. Male *Dba2/J* control and *D2.mdx* mice were treated with a single IV injection of either saline or AAV8-MD1 at 4 x 10^12^ vg/mouse at 6 weeks of age. (**A**) Representative images of collagen VI deposition in mouse diaphragms for each treatment group are shown as whole diaphragm sections (top row; scale bar: 500 µm) as well as a random close-up of each section (bottom row; scale bar: 100 µm). (**B**) Collagen VI-positive area within an entire diaphragm section was quantified and expressed as percentage of the total diaphragm section area. (**C**) Western blot analysis displaying periostin protein expression and (**D**) subsequent quantification of the level of periostin, normalized to α-tubulin, *n* = 4–5. In (**B**,**D**), data shown as ± SEM, *n* = 4–5. Statistical analysis was performed by one-way ANOVA followed by Tukey’s multiple comparisons test: *p* < 0.0001 (****), *p* < 0.001 (***), *p* < 0.001 (**), ns: not significant.

The effect of AAV8-MD1 delivery on muscle fibrosis was analyzed by assessing the expression of collagen VI, periostin, and fibronectin, all considered markers of fibrosis [23,24,25,26]. As shown in Figure 4A, the diaphragms of the *Dba2/J* control mice already showed a level of fibrosis, as has been reported previously for skeletal muscles of this mouse, but the diaphragms of the *D2.mdx* mice displayed more collagen VI deposition. The percentage of the collagen VI-positive area normalized by the total area of the diaphragm section revealed that the *Dba2/J* control mice had 32.8 ± 1.3% diaphragm collagen VI content, whereas the saline-injected *D2.mdx* mice had a significantly higher level of collagen VI content of 50.7 ± 1.2% (*p* = 0.0002) (Figure 4B). However, the mice treated with AAV8-MD1 showed a significant reduction in collagen VI deposition compared to the *D2.mdx* mice treated with saline (34.7 ± 0.9%; *p* = 0.0008). To assess the content of fibronectin and periostin, immunostaining was performed for fibronectin and Western blot analysis was performed for both proteins (Figure 4C,D and Supplementary Figure S2). The relative expression of both fibronectin and periostin proteins in *Dba2/J* mouse diaphragms was significantly lower than in the saline-treated *D2.mdx* mice. Fibronectin showed a modest, non-significant reduction by Western blot and immunostaining after AAV8-MD1 administration (Supplementary Figure S2A,B). On the contrary, the treatment with AAV8-MD1 effectively reduced the periostin protein expression by 45% (from 1.116 ± 0.060 of saline-treated mice to 0.617 ± 0.132 in AAV-treated samples) (Figure 4C,D).

### 2.5. Administration of AAV8-MD1 Leads to Downregulation of Fibrosis and Inflammation Related Genes in the Diaphragm of D2.mdx Mice

**Figure 5:**
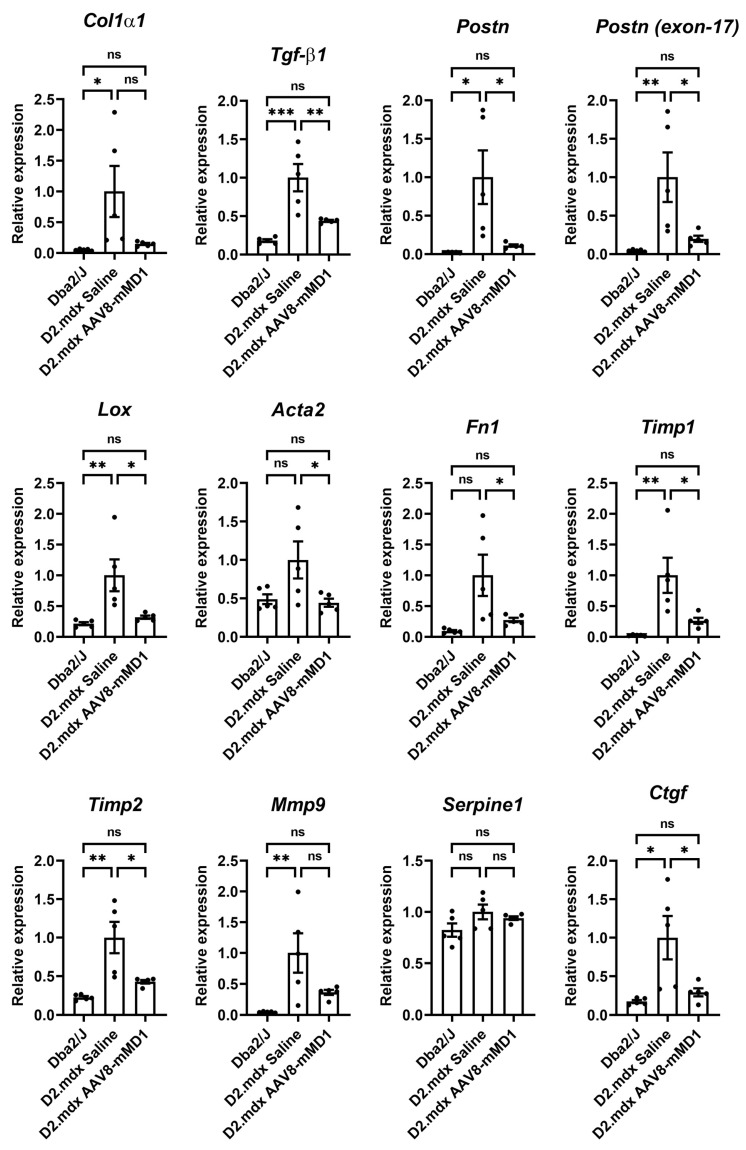
AAV8-MD1 delivery significantly reduces the mRNA expression of fibrotic markers in the diaphragms of *D2.mdx* mice. RT-qPCR analyses of the expression of downstream fibrotic genes, including *Col1α1*, *Tgf-β1*, *Postn, Postn* (exon 17-containing variants), *Lox*, *Acta2*, *Fn1*, *Timp1*, *Timp2*, *Mmp9*, *Serpine1*, and *Ctgf,* as relative to *Gapdh* expression and as fold-change compared to *D2.mdx* levels. Data shown as means ± SEM, *n* = 4–5. Statistical analysis was performed by one-way ANOVA followed by Tukey’s multiple comparisons test: *p* < 0.05 (*), *p* < 0.01 (**), *p* < 0.001 (***), *p* < 0.0001 (****), ns: not significant.

RNA extracted from the mouse diaphragms was subjected to RT-qPCR analysis to assess the effect of AAV8-MD1 treatment on the mRNA expression of downstream fibrotic genes. The gene expression was normalized to *Gapdh* to obtain a normalized relative quantity (Figure 5). Compared to the *Dba2/J* control mice, the *D2.mdx* mice in the saline-injected group exhibited significantly higher relative mRNA expression in the majority of the examined genes, including *Col1α1* (*p* = 0.04), *Tgf-β1* (*p* = 0.0003), *Postn* (*p* = 0.013), *Postn* (exon 17-containing variants) (*p* = 0.009), *Lox* (*p* = 0.008), *Acta2* (*p* = 0.072), *Fn1* (*p* = 0.017), *Timp1* (*p* = 0.004), *Timp2* (*p* = 0.002), *Mmp9* (*p* = 0.009), *Serpine1* (*p* = 0.111), and *Ctgf* (*p* = 0.011). The administration of AAV8-MD1 significantly decreased the expression of *Tgf-β1* (*p* = 0.006), *Postn* (*p* = 0.023), *Postn* (exon 17-containing variants) (*p* = 0.027), *Lox* (*p* = 0.019), *Acta2* (*p* = 0.049), *Timp1* (*p* = 0.022), *Timp2* (*p* = 0.013), and *Ctgf* (*p* = 0.027) compared to the saline-injected *D2.mdx* mice, to the levels seen in the *Dba2/J* controls.

We further performed the RT-qPCR analysis of key inflammatory markers (Supplementary Figure S3). Consistent with the findings of [10], the expression levels of all pro-inflammatory genes, including Ccl2, Cd11b, Il-6, and Tnfα, were significantly higher in the *D2.mdx* mice relative to the *Dba2/J* levels. AAV-MD1 treatment effectively downregulated the expression of Ccl2 (*p* = 0.041), Cd11b (*p* = 0.015), and Il-6 (*p* = 0.041) seen in *D2.mdx* to the levels of the *Dba2/J* controls.

## 3. Discussion

Gene therapy based on AAV-microdystrophin delivery is currently among the most promising treatments for DMD. Four clinical trials are now running in the US (3) and Europe (1) [5] with very promising results so far. Two of the trials use microdystrophin constructs including the four spectrin-like repeats 1–3 and 24, and three hinges. We have been optimizing and testing one of these microdystrophins, called MD1 [6,27,28]. Our MD1 construct is driven by the skeletal and cardiac muscle-specific promoter Spc5-12 and delivered by AAVs serotype 8. We have demonstrated previously that the AAV8-MD1 agent is highly efficient in both the mdx mouse model and in the GRMD dog model of DMD [28]. The *D2.mdx* mouse has recently been characterized in terms of disease progression and it was found that by 3 months of age, there is substantial fibrosis development in the diaphragm and gastrocnemius muscles [22]. As AAV8-MD1 has never been challenged in such a fibrotic environment, we decided to perform a systemic administration, using doses that are comparable to those currently used in clinical trials [5]. We focused our study on the diaphragm, a muscle that has not been previously analyzed in detail after the injection of gene therapy agents [20,21]. To assess the effect of AAV-MD1 on pathology, we started the experiment in 6-week-old mice, where fibrosis deposition can still be partially prevented, and we ended the experiment at 13 weeks of age, at which point the extent of the fibrosis is substantial enough to hinder muscle function. Under this timeframe, we expected that the beneficial effect of the microdystrophin gene addition therapy would be detectable using various outcome measures employed in this study.

Eight weeks of AAV8-MD1 treatment in juvenile *D2.mdx* mice effectively normalized the muscle mass, decreased fibrotic content, and improved the overall histopathology of the diaphragm muscles, rescuing the systemic muscle function in the diseased mice. As demonstrated, the absolute and specific force of TA muscle, following AAV8-MD1 administration, was normalized to that of *Dba2/J* with a frequency of stimulation of 50 Hz or more and 100 Hz or more, respectively. Similar results have also been reported in *mdx* mice treated intramuscularly with AAV9-MD1 [29]. In *D2.mdx* mice treated with AAV9-CK8-µDys-5R, Hakim and colleagues [20] showed an improvement in both absolute and specific tetanic force, though this did not reach *Dba2/J* levels. However, it is difficult to directly compare these results due to the unknown frequency of the stimulation used in the latter study. The level of microdystrophin expression in the present study also greatly improved the resistance of TA muscle to eccentric contraction-induced injury for up to six eccentric contractions, compared to the values of the saline-treated muscle. Hakim and colleagues [20] showed similar improvement; however, their study did not include *Dba2*/*J* values, thus it is not known whether such improvement was normalized to the level of *Dba2*/*J*.

The systemic administration of AAV8-MD1 at a dose of 4 × 10^12^ vg/mouse restored dystrophin expression in the diaphragm muscles to a level similar to that seen in dystrophin in *Dba2*/*J* mice. This is consistent with the microdystrophin protein expression in over 75% of the diaphragm myofibers of treated mice that led to a significant improvement in mean fiber diameter and a reduction in the number of centrally nucleated fibers in *D2.mdx* diaphragms. Together, our data highlight the functionality of microdystrophin overexpression in protecting muscle from contraction-induced turnover. AAV8 vectors incorporating the MD1 construct have previously been administered via intramuscular injection to the TA muscle of neonatal *mdx* mice and this was shown to normalize central nucleation [27]. Furthermore, the reduction in central nucleation was additionally observed with MD1 in an AAV9 vector when injected into the TA of 5-month-old *mdx* mice [29]. However, the reduction in the central nucleation we observed here is more relevant, as it was obtained by systemic delivery and in an adult mouse that also presented already substantial fibrosis at the time of the injection. Furthermore, unlike *mdx* mice, which present with muscle hypertrophy, *D2.mdx* muscles are generally atrophic, which more closely resembles the condition observed in DMD patients [10,11,30]. In this condition, we observed a reduction in the mean fiber diameter and a marked negative skew in the frequency distribution of the minimum fiber diameter of diaphragm muscle, which indicated a higher prevalence of small muscle fibers, a known characteristic of *D2.mdx* skeletal muscle [10,11,20].

The increase in the mass of the diaphragm muscle observed in the *D2.mdx* mice compared to the *Dba2/J* mice in the present study was coupled with an increased level of muscle inflammation and, consequently, increased fibrosis deposition, as evidenced by the RT-qPCR quantification of related genes and collagen VI immunostaining analysis. Previously, *D2.mdx* mice at 4 months of age have been shown to have fibrosis in the diaphragm at about ~30% [22]. This is lower than the value observed here, but this difference can be attributed to the different methods of detection used, such as decalcification prior to picrosirius red staining [22] versus immunostaining with a collagen VI-specific antibody. To measure the outcome of the treatment on fibrosis, we measured collagen VI and fibronectin expression, two established markers of fibrosis [31]. While collagen VI was clearly reduced after treatment, fibronectin expression was unaltered, highlighting the fact that our approach was beneficial, but incomplete in challenging fibrosis deposition. To further address this, we also used periostin, a matricellular protein that has been suggested to be a marker of fibrosis and tissue damage [25]. This protein is thought to have detrimental impact on fibrotic disorders [32]. While no data currently exist on periostin protein upregulation in this mouse model, it has previously been described in *Sgcg^-/-^* [33] and *mdx4-cv* [25] mice, the latter of which was coupled with collagen VI upregulation. The upregulation of *Tgf-β1* is characteristic of this mouse model [12,22], as is the upregulation of *Col1α1* and *Ctgf* [11]. We show here the upregulation of periostin at both the transcript and protein level in diaphragms from *D2.mdx* mice, providing further evidence of the role of periostin in fibrosis development in dystrophic muscle. Restoring dystrophin expression in this fibrotic DMD mouse model with AAV8-MD1 treatment leads to the downregulation of periostin protein expression in the diaphragm. In a previous study by Hakim et al. [20], 10-week-old, male, *D2.mdx* mice treated with AAV9-microdystrophin at a dose of 2 × 10^13^ vg/mouse displayed a reduction in fibrosis in the quadriceps, tibialis anterior, and diaphragm muscle, though they did not quantify the level of reduction. Here, we report a significant reduction in fibrosis with a log-fold lower dose of AAV8-microdystrophin. Our assessment of the beneficial effect of microdystrophin gene addition on the levels of fibrosis was made at the previously published peak of this phenotype in the *D2.mdx* model, rather than at six months of age, as performed by [20]. In addition to using a different AAV vector serotype and a higher dose, the microdystrophin (CK8-µDys-5R) used by Hakim and colleagues [20] employed a muscle creatine kinase-derived CK8 muscle-specific promoter to drive expression in contrast to our synthetic Spc-512 muscle-specific promoter. Furthermore, contrary to our MD1 construct, the CK8-µDys-5R microdystrophin included spectrin-like repeats 16 and 17, these being responsible for the binding of nNOS [34,35], the loss of which from the sarcolemma plays a role in DMD pathology [36,37]. The human equivalent of these two microdystrophin constructs are both being tested in clinical trials. As the results are published, it will be interesting to see the impact that the absence of the nNOS binding domain has, although direct comparisons will be difficult.

In the present study, we observed a normalization of the ratio of diaphragm mass to body mass back to wild-type levels, as well as a normalization of fiber diameter with gene addition therapy. Moreover, AAV8-MD1 administration resulted in a reduction in centrally nucleated fibers, collagen VI deposition, and of mRNA expression of fibrosis and inflammation-related genes. Finally, an improvement in the physical abilities of the mice was restored to a level that was indistinguishable from the positive *Dba2/J* controls. Using a severe mouse model of DMD, we provide further evidence of the efficiency of the MD1 construct for the restoration of dystrophin expression that dramatically improves muscle histopathology and physiology.

## 4. Materials and Methods

### 4.1. AAV8-MD1 Production

Recombinant AAV8 vectors with Spc512-MD1 transgenes were produced through the transfection of adherent HEK293T/c17 cells in Corning roller bottles with a two-plasmid system including pssAAV-Spc512-mMD1 and DP8 plasmids. AAV vectors were harvested as previously described [6] and then purified using an Akta Start HPLC machine (Cytiva, Marlborough, MA, USA) utilizing a POROS™ GoPure™ AAVX Pre-packed Column (A36648; Thermo Fisher Scientific, Bohemia, NY, USA). The virus was eluted using 0.1 M glycine (pH 2.0) followed by subsequent dialysis using a Slide-A-Lyzer™ G2 Dialysis Cassette (88252; Thermo Fisher Scientific, Bohemia, NY, USA) overnight in 1× PBS. The desalting and concentration of the viral prep was carried out using an Amicon Ultra-15 Centrifugal Filter Unit with Ultracel-100 membrane (MilliporeSigma, Burlington, MA, USA). Centrifugation was performed at 4000× *g* for 10 min. The viral vector preparation was then filter sterilized with a 13 mm syringe filter, quantified using qPCR, aliquoted, and stored at −80 °C.

### 4.2. Animals and In Vivo Experimental Design

Ethical and operational permission for in vivo experiments was granted by the Animal Welfare Committee of Royal Holloway University of London. The animal work was performed in accordance with UK government regulations and was approved on 17/12/2019 by the UK Home Office under Project License P36A9994E. D2.B10-Dmd*^mdx^*/J (referred to as *D2.mdx*) and *Dba2/J* mice were purchased from The Jackson Laboratory (Ellsworth, Maine, USA) and colonies were maintained in a minimal disease facility at Royal Holloway University of London. The mice were maintained in a standard 12 h light/dark cycle with free access to food and water. Since *D2.mdx* muscle pathology was previously studied in male mice [22], only male mice were used for the experiments.

*D2.mdx* male mice were used for this study with five mice per treatment group and four age-matched *Dba2/J* male mice included as positive controls. Six-week-old *Dba2/J* and *D2.mdx* mice received a single tail vein intravenous (IV) dose of either 4 × 10^12^ vg/mouse of AAV8-MD1 or saline. The mice were weighed on a weekly basis. The animals underwent forelimb grip strength and treadmill tests (at 5 and 12 weeks of age), after which they were placed under terminal anesthesia for in situ muscle electrophysiology followed by tissue collection (at 13 weeks of age).

### 4.3. Forelimb Grip Strength Analysis

A grip strength meter (Linton Instrumentation, Diss, Norfolk, UK) was utilized for the assessment of mouse forelimb grip strength. The maximal force was recorded as grams of force and was normalized to the body weight of the mouse at the time of the assessment. A total of five measurements were taken for each mouse with a rest period of 30 s between each reading. The lowest and highest readings for each mouse were disregarded with the three median values used for analysis. The order of the mice in the assessment was randomized each day and the assessment was carried out in a blinded manner. Assessment was performed prior to the first injection and at the conclusion of the study.

### 4.4. Fatigue Resistance Analysis

Fatigue resistance analysis was carried out using a Treadmill Simplex II (Columbus instrumentation, Columbus, Ohio, USA) with 15% inclination. The mice were allowed to acclimatize to the apparatus for 5 min prior to assessment. Following acclimatization, the run was started at 5 m/min for the first 5 min, after which the speed was increased by 0.5 m every 1 min. The exhaustion of the animals was determined by their inability to run away from the stopper after 10 secs. The assessments were performed prior to the first injection and at the conclusion of the study, with the activity being performed at the same time of day. The order of the mice was randomized at each assessment, with the treatment group being undisclosed to prevent biased results.

### 4.5. In Situ Muscle Electrophysiology

In situ muscle electrophysiology was carried out according to the TREAT-NMD SOP DMD M.2.2.005. The mice were weighed prior to assessment and were put under deep anesthesia with a mixture of 10 mg/kg dolethal (Vetoquinol UK Ltd, Towcester, UK) and 15 μg/mL buprenodale (Dechra, Veterinary Products, Shrewsbury, UK) at six times the body weight (g). The distal tendon of the tibialis anterior (TA) was isolated and the sciatic nerve stimulated using supramaximal square-wave pulses at 0.1 ms duration (701A stimulator, Aurora Scientific, Aurora, ON, Canada) and at increasing frequencies (10, 30, 40, 50, 80, 100, 120, 150, and 180 Hz) to establish the frequency–force relationship. The maximal absolute isometric tetanic force was determined by the plateau of the frequency–force relationship. To calculate the specific force, the absolute force was divided by the estimated TA cross-sectional area (CSA), which was calculated based on the formula suggested by TREAT-NMD SOP DMD_M.2.2.005. Subsequently, the TA muscle was assessed for resistance to eccentric-induced muscle damage. Eccentric contraction was induced by lengthening the muscle by 10% of its initial length and recording the maximal force after each contraction. A total of 10 eccentric contractions were performed and the data presented as eccentric torque loss as a percentage of the baseline.

### 4.6. Sample Collection and Processing

Samples were collected according to the TREAT-NMD protocol DMD_M.1.2.007. The mice were euthanized according to schedule 1 procedures. For each mouse, the diaphragm, TA, soleus, and quadriceps muscles were collected and weighed. Tissues from one side of body and half of the diaphragm were mounted in optimal cutting temperature (OCT) medium (VWR, Lutterworth, UK) and frozen in isopentane (Sigma-Aldrich, Welwyn Garden City, UK) chilled in liquid nitrogen. For the embedding of the hemi-diaphragm, it was rolled into a rosette. The remaining tissues were frozen in liquid nitrogen for protein and RNA extraction. All samples were stored at −80 °C. Frozen diaphragm samples embedded in OCT medium were cryosectioned at a thickness of 10 μm, and at 100 μm intervals using an OTF5000 cryostat (Bright Instruments, Huntingdon, UK).

### 4.7. Immunohistochemistry Staining

The diaphragm sections were air dried and fixed with 100% acetone followed by rehydration with 1× PBS thrice for 3 min. Staining was performed following the protocol of Lu-Nguyen and colleagues (2022). For collagen immunostaining, the samples were blocked in 1× PBS, 1% (*w*/*v*) BSA, 1% (*v*/*v*) goat serum, and 0.1% (*v*/*v*) Triton X-100 for 1 h at room temperature. This was followed by a primary antibody incubation with rabbit anti-collagen VI (1:300; Abcam, Cambridge UK) overnight at 4 °C. The samples were washed with 1× PBS and 0.05% (*v*/*v*) Tween-20, followed by secondary antibody incubation with goat anti-rabbit AlexaFluor488 (1:500; Invitrogen, Renfrewshire, UK) for 30 min at room temperature. The slides were mounted in Mowiol 4–88 (Sigma-Aldrich, Welwyn Garden City,, UK). The largest diaphragm sections were selected, and images were captured on an Axio Observer D1 microscope (Zeiss, Cambridge, UK) with an AxioCam MR3 at a 100× magnification. To generate images of whole tissue sections, mosaic images were captured and were automatically stitched together by ZEN imaging software (Zeiss, UK). For dystrophin and laminin immunostaining, the samples were blocked with mouse-on-mouse (MOM) blocking buffer (Vector Laboratories, UK) in 1× PBS, 1% (*w*/*v*) BSA, 1% (*v*/*v*) goat serum, and 0.1% (*v*/*v*) Triton X-100 for 1 h at room temperature. The samples were washed with 1x PBS, 0.05% (*v*/*v*) Tween-20. Primary antibody incubation was performed for 1 h at room temperature with MANEX 1011c antibody (1:50) and rabbit anti-laminin (1:500; Abcam, UK) diluted in 1× PBS supplemented with MOM diluent ( SBScientific, Kidlington, UK). The samples were washed prior to a 1 h incubation with anti-rabbit AlexaFluor488 (1:500, Invitrogen, Renfrewshire, UK). Following a wash, the samples were incubated for 10 min with anti-mouse IgG (1:250; MOM kit; Vector Laboratories, UK), washed again, and incubated for 5 min in Streptavidin AlexaFluor568 (4:250; Abcam, UK). A final incubation for 5 min with DAPI (1:1000) was performed and the slides were mounted in Mowiol 4–88 (Sigma-Aldrich, Welwyn Garden City, UK). For fibronectin and laminin co-immunostaining, the samples were blocked with MOM blocking buffer as described above. Incubation with primary antibodies, mouse anti-fibronectin F9 ascites (1:10) [38], and rabbit anti-laminin (1:500, Abcam, UK) was conducted at 4 °C overnight, followed by 1 h incubation with corresponding secondary antibodies, goat anti-mouse AlexaFluor488, and goat anti-rabbit AlexFluor568 (1:500, Invitrogen, UK). The slides were mounted in Mowiol 4–88 and the images were captured as described above.

### 4.8. Histological Analysis

Histological analysis was performed following a protocol by Lu-Nguyen and colleagues [26]. Collagen VI immunostaining was used to determine the fibrotic area of each sample. A semi-automated analysis using Fiji/MuscleJ software (National Institutes of Health, Maryland, USA) was utilized to determine the total CSA and collagen VI-positive area of the sample, which was used to calculate the fibrotic area as a percentage of the CSA. Laminin immunostaining was used for the total fiber count and the minimal Feret’s diameter of each individual fiber. These data were acquired automatically by Fiji/MuscleJ software (National Institutes of Health, Bethesda, Maryland, USA). The frequency distribution of the minimal Feret’s diameter was achieved by an automated analysis using GraphPad Prism 9 software (GraphPad, San Diego, CA, USA). Laminin and DAPI immunostaining allowed for the assessment of centrally nucleated fibers (CNFs), which were measured automatically by Fiji/MuscleJ software and expressed as a percentage of the total fiber count. Dystrophin staining was used to calculate the amount of dystrophin-positive fibers. For each whole diaphragm section, the number of dystrophin-positive fibers (those that were positive for dystrophin around the whole perimeter of the fiber) was counted manually and expressed as a percentage of the total fiber number automatically counted. All histological analyses were performed with the treatment groups being undisclosed to prevent biased results. Hematoxylin and eosin staining was performed using an Abcam H&E staining kit (ab245880; Abcam, Boston USA). Images of the muscle sections were acquired using an Olympus VS120 microscope (Olympus, Center Valley, PA, USA) at a 40× magnification.

### 4.9. RNA Extraction from Tissues, cDNA Synthesis, and Quantitative Polymerase Chain Reaction

Muscle tissue (30 gs) was homogenized in RLT buffer using a 3 mm Tungsten carbide bead (69997; QIAGEN, Hilden, Germany) and TissueLyser II (QIAGEN, Hilden, Germany) at 25 hz for 4 min with a rotation of tubes after 2 min. The samples were processed using the RNeasy fibrous tissue mini kit (QIAGEN, Hilden, Germany) following the protocol set out by the manufacturer. The concentration of RNA was determined using a Nanodrop ND-1000 spectrophotometer. The OD260/280 ratio given by the spectrophotometer assessed the purity of the nucleic acids, with pure nucleic acids having an OD260/280 ratio between 1.8 and 2.0.

cDNA synthesis was performed using the QuantiTect Reverse Transcription Kit (QIAGEN, Hilden, Germany). Then, 1000 ng RNA was mixed with 2 µL genomic DNA wipeout buffer and made up to 14 µL with water. This was incubated for 2 min at 42 °C, followed by the addition of 4 µL of 5× Quantiscript RT buffer, 1 µL RT primer mix, and 1 uL Quantiscript Reverse Transcriptase, to obtain a final volume of 20 µL per reaction. This reaction mix was then incubated for 15 min at 42 °C followed by 3 min at 95 °C.

qPCR was performed using the optimized primers detailed in Supplementary Table S1 using the LightCycler480 system (Roche, Mannheim, Germany). The standards were serially diluted from 10^7^ to 10^1^ copies per 2 µL. This was then added to 8 µL PCR mix containing 1x SYBR Green (Roche, Mannheim, Germany), 0.4 µL of each 10 µM primer (final concentration of 400 nM), and 2.2 µL qPCR-grade water (Roche, Mannheim, Germany). Then, 2 µL of diluted cDNA (dilution factor dependent on the abundancy of the gene of interest) was also mixed with 8 µL PCR mix, as above. The qPCR program used is as follows; polymerase activation at 95 °C for 5 min; 45 cycles of denaturation at 95 °C, annealing at 57–60 °C, and extension at 72 °C, each for 15 s; melt curve at 95 °C for 5 s, 65 °C for 60 s, and a final 97 °C melt curve and hold. Data analysis was performed using the LightCycler480 software (Roche, Mannheim, Germany). For the relative quantification of the gene of interest, the samples were normalized to the levels of Glyceraldehyde 3-phosphate dehydrogenase (*Gapdh*).

### 4.10. Protein Extraction from Tissue, Protein Assay, and Western Blot

A 3 mm Tungsten carbide bead (69997; QIAGEN, Hilden, Germany) was placed in an Eppendorf tube containing 30 mg of tissue sample in 300 µL RIPA lysis buffer and homogenized using TissueLyser II (QIAGEN, Hilden, Germany) at 25 hz for 4 min, with the rotation of tubes after 2 min. The samples were centrifuged at 14,000× *g* for 10 min at 4 °C. The supernatant (protein extract) was decanted to a fresh, pre-chilled Eppendorf tube and the pellet was discarded. The total protein content of the sample was measured using the DC Protein Assay (Bio-Rad, Watford, Hertfordshire, UK) following the manufacturer’s standard protocol. Then, 10 to 25 µg total protein was mixed with 2 µL reducing agent (Li-Cor, Lincoln, NE USA), 5 µL of 4× lithium dodecyl sulfate (Li-Cor, Lincoln, USA), and made up to 20 µL with water. The samples were denatured at 70 °C for 10 min. For the dystrophin Western blot, the mouse monoclonal MANEX1011c (DSHB Hybridoma bank, Iowa, UK) 1:50 was used. For the periostin Western blot, Anti-periostin/OSF-2 Isoform 2 antibody (AF2955; R&D Biosystems, Minneapolis, Minnesota, USA) and the housekeeping anti-alpha tubulin antibody (ab4074; Abcam, Cambridge, UK) were used. For the fibronectin Western blot, the membrane following periostin and alpha-tubulin immunoblotting was stripped with stripping buffer (1.5% (*w*/*v*) glycine, 1% (*w*/*v*) SDS, 1% (*v*/*v*) Tween-20, pH 2.2) and reincubated with anti-fibronectin F9 ascites antibody [38]. For blocking, the Li-Cor blocking buffer was used. Secondary antibodies were obtained from Li-Cor. The membranes were visualized using the Odyssey CLx system (Li-Cor, Lincoln, USA) and analyzed using Image Studio software.

### 4.11. Statistical Analyses

Statistical analysis was performed using GraphPad Prism 9 software (California, USA) and shown as means ± SEM. The normality of the data was assessed using a Shapiro–Wilk test, with the distribution being assumed normal if the *p* value was ≥0.05. The homogeneity of variance was assessed using the Brown–Forsythe test, with the data being assumed homoscedastic if the *p* value was ≥0.05, or heteroscedastic if the *p* value was ≤0.05.

For comparisons between two groups, a two-tailed t-test was performed. For comparisons among multiple groups, if the conditions of normality and homoscedasticity were met, a one-way ANOVA followed by a Tukey’s multiple comparisons test was performed. In the case of normally distributed, heteroscedastic data, a Welch’s ANOVA followed by a Dunnett’s T3 multiple comparisons test was performed. If the condition of normality was not met in terms of homoscedastic distribution, a Kruskal–Wallis ANOVA followed by a Dunn’s multiple comparisons test was performed. Testing for outliers was performed using a Grubbs’ test (Alpha = 0.05).

## Data Availability

The data presented in this study are available on request from the corresponding author. The data are not publicly available due to further development of the work.

## Supplementary Materials

The following supporting information can be downloaded at: https://www.mdpi.com/article/10.3390/ijms24098174/s1.
