# Supplementary material for: Microdystrophin Gene Addition Significantly Improves Muscle Functionality and Diaphragm Muscle Histopathology in a Fibrotic Mouse Model of Duchenne Muscular Dystrophy
Source: Int J Mol Sci. 2023 May 3;24(9):8174. doi: 10.3390/ijms24098174 (PMC10179398; doi:10.3390/ijms24098174)
Supplement: Supplementary file 1 [file ijms-24-08174-s001.zip › ijms-2347002-supplementary.pdf]

Supplementary Materials

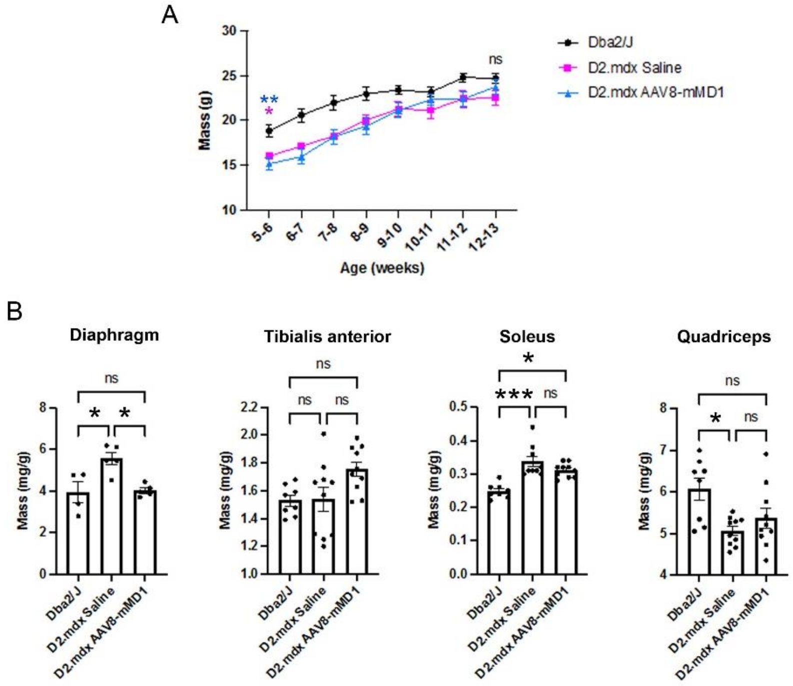

Figure S1. Effect of AAV8-MD1 on body and muscle mass of *D2.mdx* mice. Male *Dba2/J* control mice and *D2.mdx* treatment groups were weighed weekly starting from 6 weeks of age. Mice at 6 weeks of age were subsequently treated with a single IV injection of either saline or AAV8-MD1 at 4×10^{12} vg/mouse. Muscles were weighed when harvested at the end of the experiment. (**A**) The weekly mean body mass per group shows that while the body mass at the beginning of the experiment was significantly different between *Dba2/J* and *D2.mdx* mice, at the end of the treatment (13 weeks of age) all mice had a similar weight. Data shown as means ± S.E.M., n = 4–5 mice. Statistical analysis was performed by a two-way ANOVA followed by a Tukey's multiple comparisons test, * $p < 0.05$ (*Dba2/J* vs saline treated *D2.mdx)*, ** $p < 0.01$ (*Dba2/J* vs AAV8-MD1 treated *D2.mdx*). (**B**) At 14 weeks of age, mice were placed under terminal anesthesia and the diaphragm, tibialis anterior, soleus, and quadriceps muscles were harvested and muscle mass was normalized to corresponding body mass (mg/g). No substantial change in muscle mass was detected for skeletal muscles except for the diaphragm muscle. Data shown as means ± S.E.M., n = 4–5 mice. Statistical analysis was performed using a one-way ANOVA followed by a Tukey's multiple comparisons test, $p < 0.05$ (*), $p < 0.01$ (**), $p < 0.001$ (***), ns: no significant difference was observed.

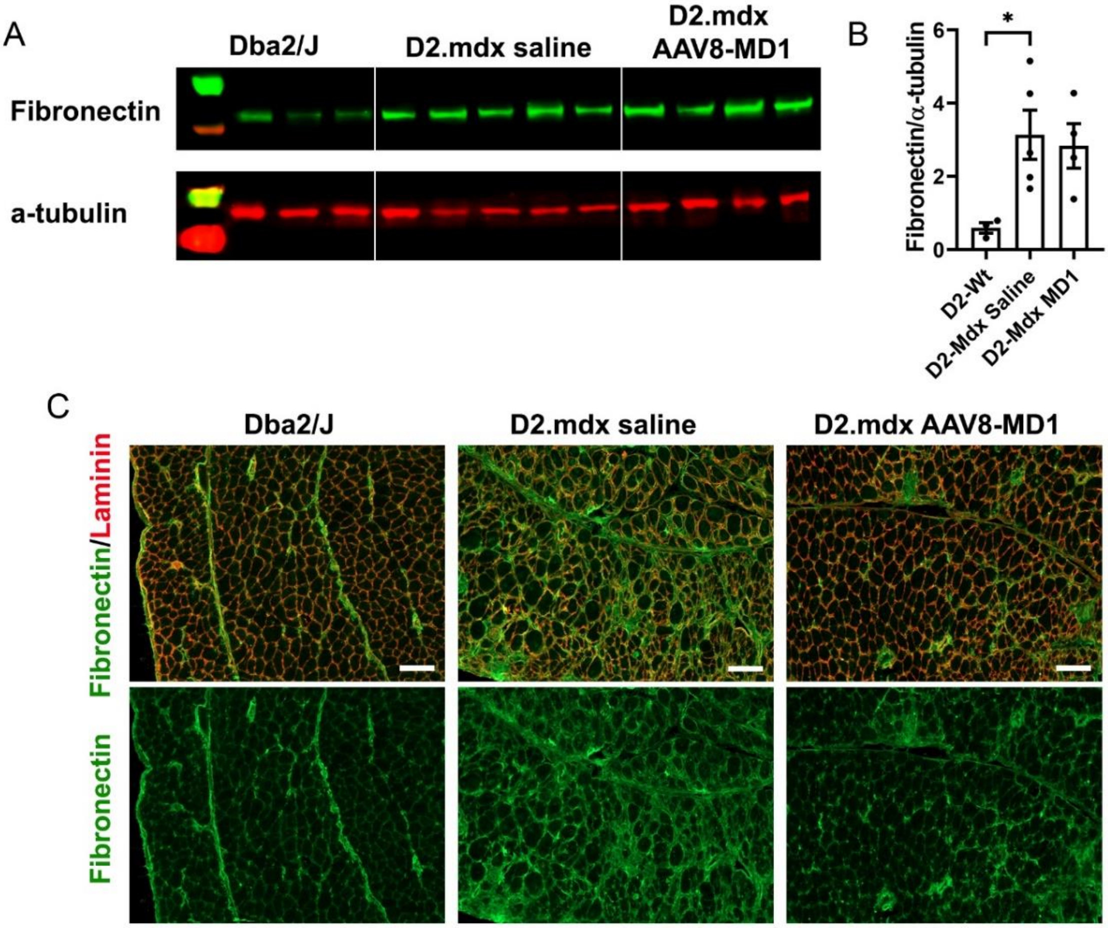

Figure S2. Fibronectin expression shows a minimal reduction in treated mice. Male *Dba2/J* control and *D2.mdx* mice were treated with a single IV injection of either saline or AAV8-MD1 at 4×10^{12} vg/mouse at 6 weeks of age. (**A**) Western blot analysis displaying fibronectin protein expression shows an increase in protein expression in *D2.mdx* mice compared to *Dba2/J* mice but no reduction after AAV treatment. (**B**) subsequent quantification of the level of fibronectin, normalised to α-tubulin, n = 4–5. (**C**) Representative images of fibronectin deposition in mouse diaphragms for each treatment group. Laminin staining is in red. Scale bar: 100 µm). Statistical analysis was performed by a one-way ANOVA followed by a Tukey's multiple comparisons test, $p < 0.05$ (*).

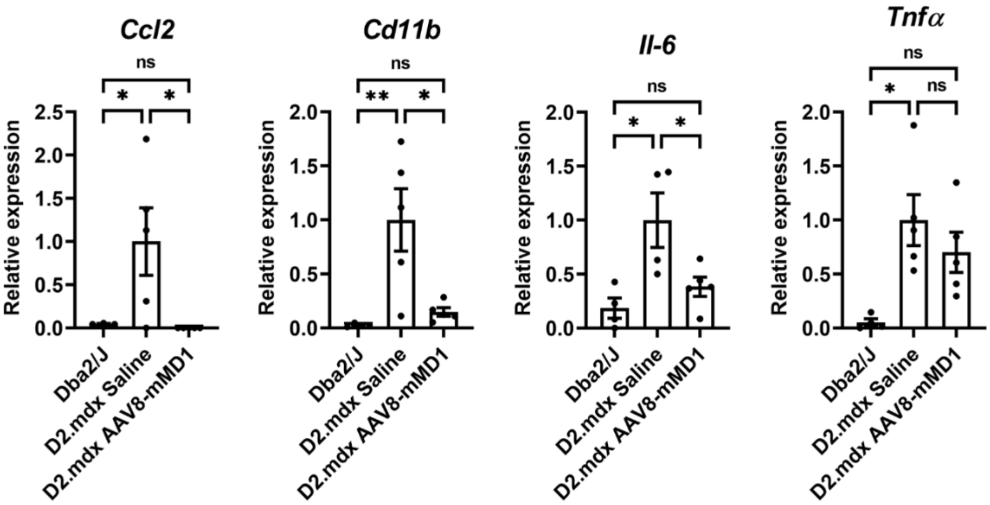

Figure S3. qPCR for inflammatory markers. qPCRs performed for Ccl2, Cd11b, IL-6 and Tnf-α show a general substantial reduction of the first 3 markers after treatment, while Tnf-α expression remained unaltered. Statistical analysis was performed by a one-way ANOVA followed by a Tukey's multiple comparisons test, $p < 0.05$ (*), $p < 0.01$ (**), ns: not significant.

Table S1. qPCR primer sequences.

Target genes (mouse)	Primer sequence 5′–3′	PCR product size (bp)
Alpha actin 2 (*Acta2*)	Forward primer: 5′-GAGCTACGAACTGCCTGAC-3′<br>Reverse primer: 5′-CTGTTATAGGTGGTTTCGTGGA-3′	129
Fibronectin 1 (*Fn1*)	Forward primer: 5′-GAGCTATCCATTTCACCTTCAGA-3′<br>Reverse primer: 5′-TTGTTCGTAGACACTGGAGAC-3′	96
Periostin (*Postn*)	Forward primer: 5′-CCTGTAAGAACTGGTATCAAGGT-3′<br>Reverse primer: 5′-CCTTTCATCCCTTCCATTCTCA-3′	97
Tissue inhibitor metalloproteinase 1 (*Timp1*)	Forward primer: 5′-AGACAGCCTTCTGCAACTC-3′<br>Reverse primer:5′-CAGCCTTGAATCCTTTTAGCATC-3′	129
Tissue inhibitor metalloproteinase 2 (*Timp2*)	Forward primer: 5′-GACCTGACAAAGACATCGAGT-3′<br>Reverse primer: 5′-GCCATCTCCTTCTGCCTTT-3′	119
Ribosomal protein lateral stalk subunit P0 (*Rplp0*)	Forward primer: 5′-TTATAACCCTGAAGTGCTCGA-3′<br>Reverse primer: 5′-CGCTTGTACCCATTGATGATG-3′	147
Matrix metalloproteinases 9 (*Mmp9*)	Forward primer: 5′-GACATAGACGGCATCCAGTATC -3′<br>Reverse primer: 5′-GTGGGAGGTATAGTGGGACA -3′	125
Periostin exon 17 (*Postn-17*)	Forward primer: 5′-ATAACCAAAGTCGTGGAACCAA-3′<br>Reverse primer: 5′-CTTCCGTTTTGATAATAGGCTGAA- 3′	70
Lysyl oxidase (*Lox*)	Forward primer: 5′-ACGCTGTGACATTCGCTACA-3′<br>Reverse primer: 5′-TGTCCAAACACCAGGTACGG-3′	115
Collagen, type I, alpha 1 (*Col1a1*)	Forward Primer: 5′-GAAACTTTGCTTCCCAGATGTC-3′<br>Reverse Primer: 5′-AGACCACGAGGACCAGAA-3′	94
Serine (or cysteine) peptidase inhibitor, clade E, member 1 (*Serpine1*)	Forward Primer: 5′-AGAGGTTAAGGAAGAGGGTCCA-3′<br>Reverse Primer: 5′-TTTTCCATTGTCCATCGGCG-3′	128
Connective tissue growth factor (*Ctgf*)	Forward Primer: 5′-GTGCACTGCCAAAGATGGTG-3′<br>Reverse Primer: 5′-CTTTGGAAGGACTCACCGCT-3′	72
Transforming Growth factor β1 (*Tgf-β1*)	Forward primer: 5′-CTGCTGACCCCCACTGATAC-3′<br>Reverse primer: 5′-GCCCTGTATTCCGTCTCCTT-3′	93
Glyceraldehyde 3- phosphate dehydrogenase (*Gapdh*)	Forward primer: 5′-GTGGAGTCATACTGGAACATGTAG- 3′<br>Reverse primer: 5′-AATGGTGAAGGTCGGTGTG-3′	150
Tumour Necrosis Factor alpha (Tnfα)	Forward primer: 5 -AGCCGATGGGTTGTACCTTG-3′<br>Reverse primer: 5′-ATAGCAAATCGGCTGACGGT-3′	99
Interleukin 6 (*Il-6*)	Forward primer: 5′-CCCCAATTTCCAATGCTCTCC -3′<br>Reverse primer: 5′-CGCACTAGGTTTGCCGAGTA -3′	141
Chemokine (C-C motif) ligand 2 (*Ccl2*)	Forward primer: 5′- CCACAACCACCTCAAGCACT -3′<br>Reverse primer: 5′- AGGCATCACAGTCCGAGTCA -3′	75
Integrin alpha M (*Cd11b*)	Forward primer: 5′- AGTGCTGGGAGACGTGAATG -3′<br>Reverse primer: 5′- GCACTGAGGCTGGCTATTGA -3′	120
